# “I don’t see an added value for myself”: a qualitative study exploring the social cognitive variables associated with influenza vaccination of Belgian, Dutch and German healthcare personnel

**DOI:** 10.1186/1471-2458-14-407

**Published:** 2014-04-28

**Authors:** Birthe A Lehmann, Robert AC Ruiter, Sabine Wicker, Dick van Dam, Gerjo Kok

**Affiliations:** 1 Department of Work & Social Psychology, Faculty of Psychology and Neuroscience, Maastricht University, PO Box 616, 6200 MD Maastricht, The Netherlands; 2 Betriebsärztlicher Dienst, Klinikum der Johann Wolfgang Goethe-Universität Frankfurt, Theodor-Stern-Kai 7, 60590 Frankfurt, Germany; 3 Microbiology, Orbis Medisch en Zorgconcern, Postbus 5500, 6130 MB Sittard, The Netherlands

**Keywords:** Influenza vaccination, Healthcare personnel, Hospital, Qualitative research, Social cognitive determinants

## Abstract

**Background:**

Health Authorities recommend influenza vaccination of healthcare personnel (HCP) to decrease the transmission of influenza to vulnerable patients. Recent studies have almost exclusively used quantitative questionnaires in order to identify determinants of vaccination behaviour. Interviews enable HCP to express freely why they think they are (not) willing to get vaccinated against influenza.

**Methods:**

By means of semi-structured one-on-one interviews with 123 Belgian, Dutch and German HCP, reasons for and against vaccination, experiences with influenza vaccination, intention to get vaccinated and possible barriers, as well as willingness to advice influenza vaccination to patients were investigated. Data were processed with QSR NVivo 8.0 and analysed using a combination of a deductive and a general inductive approach.

**Results:**

Across countries, self-protection, patient protection, and protection of family members were reported as most important reasons to get vaccinated against influenza. Reasons to not get vaccinated against influenza were fear of side effects caused by the vaccine, a low risk-perception, the disbelief in the effectiveness of influenza vaccination, organizational barriers, misconceptions, and undefined negative emotions.

**Conclusions:**

The social cognitive variables underlying the decision of HCP to get vaccinated against influenza (or not) seem to be similar in Belgium, Germany, and the Netherlands, even though some differences surfaced. A quantitative investigation of those social cognitive variables is needed in order to determine the importance of the social cognitive variables in explaining the intention to get vaccinated and the importance of the similarities and differences between countries that have been found in this study.

## Background

Annual influenza epidemics are a public health problem resulting in up to five million cases of severe illness worldwide, of which 5 to 10% result into deaths each year [1]. Affected are especially children, the elderly and patient groups with specific health conditions [2,3]. Health Authorities recommend influenza vaccination of healthcare personnel (HCP) to decrease the transmission of influenza to vulnerable patients [1,4]. Studies have shown that vaccination of HCP can decrease clinical disease in healthy adults by 70-90% and might decrease all-cause mortality by 29% [2,5]. Moreover, less influenza infections among HCP has, amongst other economic benefits, the advantage of less illness-related work-absenteeism within this group [6,7].

Influenza vaccination has been shown to be safe and effective [7,8] and can be given relatively effortless to a large group of people. Although benefits are clearly demonstrated [6,7] and hospitals simplified the process of HCP getting vaccinated by offering free vaccine on work-site and by giving necessary information [9-11], the actual vaccination numbers are generally low and stay far below Health Authority recommendations [2,12,13]. A study comparing 11 European countries found vaccination coverage rates of 6.4 to 26.3% among HCP [14].

Next to the professional responsibility of HCP to get vaccinated against influenza in order to protect their patients [15], HCP and medical practitioners in particular are expected to advise influenza vaccination to their patients. However, HCP do not always advise vaccination to their patients [16].

Four reviews explored the social cognitive reasons reported by HCP for (not) getting vaccinated against influenza [17-20]. The most common reasons for rejection are a low risk-perception, doubts about the effectiveness of vaccines, fear of side-effects, and the belief that influenza is not a serious illness. The most common reasons in favour of influenza vaccination are self-protection and the belief in the effectiveness of the vaccine. Older age and previous receipt of influenza vaccination were additionally shown to predict the intention to get vaccinated [17-20].

Recent studies have almost exclusively used quantitative questionnaires in order to identify determinants of vaccination behaviour [17-20]. In these questionnaires, HCP receive constructed reasons against and in favour of influenza vaccination and are forced to choose. This may lead HCP to select answers even if they do not reflect their true reasons for (not) getting vaccinated. As was proposed by Fishbein and Ajzen [21], social cognitive variables and their underlying behavioral, normative and control beliefs should be elicited by asking people directly about them. This gives HCP the opportunity to express without restriction why they think they are (not) willing to get vaccinated against influenza. The study was conducted in three European countries, one of them not having been systematically surveyed before (Belgium). In this study, one-to-one interviews with HCP of hospitals in Belgium, Germany and the Netherlands were used in order to gain a direct and more in-depth understanding of the beliefs underlying the decision to get vaccinated against influenza of HCP that are already known, as well as allowing for the possibility to identify beliefs that have not been captured by previous quantitative studies.

## Methods

### Participants and procedure

Three hospitals participated in this study: the University Hospital Antwerp, in Belgium, the University Hospital Frankfurt, in Germany, and the Orbis Medisch Centrum, in the Netherlands. These three hospitals were chosen, because of existing contacts to either the occupational physician or the clinical microbiologist, the existence of comparable vaccination programs, and having a substantial number of the HCP not taking the vaccination (see Table 1 for distribution of immunizers and non-immunizers). All three hospitals have an annual vaccination program in which employees are encouraged to get vaccinated against influenza, they are informed about influenza vaccination and offered vaccination for free and during their working hours. However, these programs are not based on extensive social psychological investigations of the reasons HCP have for getting vaccinated or not. Possible participants were approached during their lunch breaks or in the waiting room of the occupational physician. Participation was open to all HCP. Particular effort was done to obtain a comparable proportion of physicians and nursing staff among employees from the three hospitals. Participants were provided with information concerning the purpose of the interview, anonymity and confidentiality conditions, and the voluntariness of participation before each interview. Participants were asked for permission to tape-record the interviews. Informed written consent was acquired. Interviewees were HCP from different wards and with different professions. Recruitment was performed by the first author and continued until saturation occurred [22]. Due to time constraints on the side of the HCP, interviews were short and lasted approximately ten minutes. The interviewer (BAL) had no dual relationship with the interviewees. The Research Ethics Board of the Faculty of Psychology and Neuroscience at Maastricht University reviewed and approved the study.

**Table 1:** Sample demographics and characteristics of German, Dutch and Belgian healthcare personnel

	**Total (N = 123)**	**German HCP (N = 31)**	**Dutch HCP (N = 45)**	**Belgian HCP (N = 47)**
Immunizers	50 (41%)	15 (48%)	9 (20%)	26 (55%)
Non-immunizers	73 (59%)	16 (52%)	36 (80%)	21 (45%)
Gender				
Male	40 (32%)	9 (29%)	18 (40%)	13 (28%)
Female	83 (68%)	22 (71%)	27 (60%)	34 (72%)
Mean age (SD)	37,11 (11,32)	32,77 (8,39)	36,64 (11,64)	40,40 (11,85)
Occupation				
Physician	32 (26%)	6 (19%)	19 (42%)	7 (15%)
Nurse	57 (46%)	14 (45%)	24 (53%)	19 (40%)
Student	7 (6%)	5 (17%)	0	2 (5%)
Others	27 (22%)	6 (19%)	2 (5%)	19 (40%)

*Note*: SD, standard deviation; HCP, healthcare personnel.

### Data collection and analysis

Data were collected by means of semi-structured interviews. Questions covered the topics a) general information (i.e., What is your position in this hospital?), b) immunization status and reasons for vaccination (i.e., Did you get vaccinated against influenza in the past season?; What are the reasons why you did (not) get vaccinated against influenza in the past season?), c) experiences with influenza vaccination (i.e., What are your experiences with influenza vaccination?), d) intention to get vaccinated (i.e., Are you planning to get vaccinated against influenza in the influenza season 2012/13?), and e) patient advice (i.e., Would you recommend influenza vaccination to your patients and why?). Data were processed with QSR NVivo 8.0 (Doncaster, Australia). The content analysis was based on a combination of a deductive and a general inductive approach [23]. The deductive analysis was based on concepts of the Reasoned Action Approach [21]. Content analysis was conducted by a single coder. No formal testing of reliability could be performed. However, the authors discussed and agreed on the interpretation of the data. Following analysis, quotes were selected on the basis of their representativeness for the findings and subsequently translated from German and Dutch into English. This qualitative study adheres to the RATS guidelines for reporting qualitative studies (see Additional file 1 for the RATS checklist) [24].

## Results

Interviews were completed with 47 Belgian HCP of which 26 were vaccinated (10 men and 16 women) and 21 were not vaccinated against influenza (3 men and 18 women), 45 Dutch HCP of which 9 were vaccinated (4 men and 5 women) and 36 were not vaccinated against influenza (14 men and 22 women), and 31 German HCP of which 15 were vaccinated against influenza (6 men and 9 women) and 16 were not vaccinated against influenza (3 men and 13 women; see Table 1). Inspection of the individual wards participating HCP were working on did not show any noticeable distinction with regard to vaccination status of HCP working on high risk vs. low risk wards. In the following, we will refer to the vaccinated participants as immunizers and the unvaccinated as non-immunizers. Results for immunizers and non-immunizers are described separately to detect differences between those two groups, in combination with similarities and differences among HCP from different countries. See Table 2 for a detailed overview of the given answers that are summarized below.

**Table 2:** Detailed overview of HCP answers, grouped into similarities and differences among HCP from different countries

**Topic**	**Immunizers**		**Non-immunizers**	
	***Similarities***	***Differences***	***Similarities***	***Differences***
**Reasons to (not) get vaccinated**	Self-protection; patient protection; family protection German & Belgian HCP: Pressure Dutch & Belgian HCP: Medical condition	German HCP: Having to wear a mask Belgian HCP: Vulnerable people to care for; protection of colleagues; preventing work-absenteeism; pregnancy; flu shot is free of charge	Fear of side-effects (own experience + other’s reports); (almost) Never had the flu; feeling healthy; no active consideration (emotional decision-making); forgetfulness German & Dutch HCP: Absence; time issues; disbelief in effectiveness flu shot; pregnancy German & Belgian HCP: Still possible to get ill when vaccinated Dutch & Belgian HCP: Body can deal with flu *[mostly combinations of reasons]*	German HCP: Belief in other prevention means (hand hygiene); no (close) patient contact Dutch HCP: Hospital wants to prevent work-absenteeism; annual vaccination too often Belgian HCP: Afraid of needles
**Attitude**	Mostly positive attitude	Belgian HCP: One negative attitude (does it because of felt pressure)	Dutch & Belgian HCP: Mostly positive attitude (acknowledging importance for high risk-group); Several negative attitude; some ambivalent	German HCP: Mostly neutral/ambivalent attitude (acknowledging importance for high risk-group);
**Outcome expectations**	*Advantages:* Prevention of influenza infection; weaken symptoms in case of flu or cold; prevention of transmission to patients, family members and colleagues Dutch & Belgian HCP: Less work absenteeism *Disadvantages:* Side-effects; serious risks; some none	*Advantages:* German HCP: Herd immunity	*Advantages:* Prevention of influenza infection; patient protection; some none German & Dutch HCP: advantageous for others Dutch & Belgian HCP: less work-absenteeism	*Advantages:* German HCP: No mask necessary
		*Disadvantages:* German HCP: Flu from vaccine Belgian HCP: Time-consuming		
				*Disadvantages:* Dutch HCP: Own antibodies cannot develop; time-consuming
			*Disadvantages:* Getting ill from the flu shot (side-effects/flu); uncertainty German & Belgian HCP: developing resistance to vaccine	
**Subjective norm**	German & Belgian HCP: Most talk to colleagues about vaccination; impression that most do (not) get vaccinated divided Most talk to family about vaccination; recommend it when risk group	Dutch HCP: Most do not talk to colleagues about vaccination; impression that majority also get vaccinated; decision dependent on engagement & motivation Belgian HCP: some do not talk to family about vaccination	Dutch & Belgian HCP: Many do not talk to colleagues about vaccination, or superficially Most have impression that their colleagues do not get vaccinated either German & Dutch HCP: (not) Talking to family is divided equally Many recommend it when risk group	German HCP: (not) Talking to colleagues is divided Dutch HCP: Some against vaccination of family members Belgian HCP: Most talk to family members about vaccination (do not get vaccinated either)
**Perceived behavioural control**	Free choice for most German & Belgian HCP: Some see it as a less free choice because of pressure Dutch & Belgian HCP: Moral responsibility	German HCP: One feels forced	Free choice for majority Dutch & Belgian HCP: important that it stays free	German HCP: Some see it as a less free choice because of their profession; few feel forced Dutch HCP: one sees it as a less free choice because of responsibility for patients Belgian HCP: only high risk groups cannot freely decide; or has to be social necessity
**Responsibility**	Majority feels responsible as HCP to protect patients by getting vaccinated	German HCP: Other means of protection show also responsibility Dutch HCP: Rather self-protection Belgian HCP: Should be mutual; no patient contact; unsure	Most do not think it is HCP responsibility to protect patients by getting vaccinated, hand disinfection and staying at home when ill instead; vaccination no guarantee; some feel responsible Dutch & Belgian HCP: Protection should be mutual	German HCP: Personal decision; not thought about before Dutch HCP: Physicians should be good examples; would be mandatory if it would be their responsibility; still susceptible to other bacteria/viruses
**Experiences with the flu shot**	Majority had positive experiences with the flu shot; several had pain at site of injection or local swelling Dutch & Belgian HCP: mild side-effects	German HCP: bronchitis after vaccination	Several never had flu shot before, but heard a lot from colleagues; some had side-effects German & Dutch HCP: several had positive experiences	Dutch HCP: No difference with vs. without flu shot
**Intention**	Majority intend to get vaccinated again German & Belgian HCP: Few unsure		Most do not intend to get vaccinated; some do German & Dutch HCP: unsure/doubts about necessity	German HCP: More who intend to get vaccinated compared to Belgian & Dutch HCP
**Possible barriers**	Organizational issues (time + administration); none; pregnancy Dutch & Belgian HCP: Being ill/having flu	German HCP: Fear of side-effects; long-lasting illness	Organizational issues; forgetfulness	German HCP: None; side-effects; being ill
**Patient advice**	Several would recommend vaccination to patients (belonging to the high risk group); free choice of patients German & Belgian HCP: Some would recommend vaccination to patients; few not because of time constraints/practitioner’s task	German HCP: Recommendation only if patients ask about it; lack of knowledge	Many would only recommend vaccination to patients (belonging to the high risk group) German & Dutch HCP: Some would not German & Belgian HCP: Few unsure Dutch & Belgian HCP: Practitioner’s task	German HCP: Patients should not get vaccinated; other vaccinations more important Dutch HCP: generally no recommendations; wanting to be neutral Belgian HCP: Practitioners sometimes also do not recommend it

### Social cognitive reasons

#### ***Reasons of immunizers to get vaccinated***

Belgian, Dutch and German immunizers all reported self-protection, patient protection and protection of family members as most important reasons to get vaccinated against influenza. Across the different countries, self-protection was reported most often.

“First of all to protect myself and to protect my family, that I don’t take germs home. But of course also to protect patients […]”.

Other mentioned reasons that were similar across countries were feelings of pressure to get vaccinated from the occupational physician, their employer, the head of the department, or generally the ward they were working on.

“[…] the occupational physician basically insists on doing it. It is strongly recommended to do it. It is voluntary but you are explicitly made attentive that it would be necessary”.

Less often reported reasons were having a medical condition that required annual vaccination, having to wear an FFP2 mask at work, having to care for small children or the elderly at home, the protection of fellow colleagues, prevention of work-absenteeism and being pregnant.

#### ***Reasons of non-immunizers to not get vaccinated***

In contrast to immunizers, Belgian, Dutch and German non-immunizers’ reasons for not getting vaccinated against influenza can be represented by six categories: Firstly, HCP who did not get vaccinated the past season, mostly reported that they were afraid of side-effects that the flu shot might have, in particular flu-like symptoms. Some of these fears came from own experience and some came from experiences that others had reported.

“Well, I got vaccinated before and then I was ill with the flu. I had to stay at home for two weeks with real flu symptoms. And since then I said ‘No, I don’t want it anymore’.”

Secondly, non-immunizers reported that they did not feel at risk to get the flu since they never or almost never had the flu before and reported to feel healthy.

“I never got vaccinated (against influenza) and I never had the flu. So, yeah”.

Thirdly, the disbelief in the effectiveness of influenza vaccination in protecting themselves or patients was reported as a reason to not get vaccinated.

“I’m uncertain whether it (influenza vaccination) is working or not”.

The fourth category of reasons to not get vaccinated against influenza comprised organizational barriers.

“[…] the reason I didn’t do it this season was that the appointments were set on weird times and then I couldn’t make it […]”

Lack of knowledge and misconception about who should get vaccinated, the belief that a person benefits from undergoing illness and that there are other protective measures that are more effective in preventing the flu were mentioned as a fifth category.

“[…] And yes, maybe I also thought that because of the pregnancy, I wasn’t really sure about it (flu shot), even though the occupational physician said it (that it is recommended) […]”

Finally, several non-immunizers reported undefined negative emotions or fear resulting from the decision whether to get vaccinated.

“I do think about it, but in the end it’s a calculation of – I don’t know what – rather emotional arguments that tell you, okay it is going well without it (flu shot)”.

#### ***Attitude and outcome expectations of immunizers***

Most immunizers reported a positive attitude towards influenza vaccination. Positive outcome expectations originated from the belief in the effectiveness of the flu shot to prevent infections and transmission of influenza to patients and family members.

“Advantages… I mainly see them for my patients. That I won’t transmit flu. So actually as a protection for others. For me personally, it doesn’t really make a difference. At my age”.

Additionally, creating herd-immunity, but also preventing work-absenteeism was seen as an advantage.

”I can’t permit getting ill with my job. So if that can be prevented, why not?”

Negative outcome expectations were possible side-effects or more serious consequences that might result from vaccination, but also the local pain that can be associated with an injection.

“The flu shot can have side effects. I experienced it myself that you feel ill immediately after vaccination, feeling shivery”.

#### ***Attitudes and outcome expectations of non-immunizers***

Among the non-immunizers, Belgian and Dutch HCP reported a positive attitude towards influenza vaccination, while German HCP were rather ambivalent. All non-immunizers acknowledged the importance of influenza vaccination for high risk groups.

“I do have a positive attitude towards it (influenza vaccination). For people that are as healthy as I am, it’s not necessary. But of course for babies and the elderly, or women who are pregnant, or people who are really ill, it is beneficial”.

Positive and negative outcome expectations resembled those of the immunizers. However, additional disadvantages were mentioned to be the development of a resistance to the vaccine and that there would be no guarantee for the effectiveness of the flu shot because of annual mutations of the influenza virus.

“[…] you never know how the substance itself will affect you. How you tolerate it and you also don’t know when you get vaccinated against the flu – there are always different types every year - if this, exactly this type is included in the vaccine”.

#### ***Subjective norms of immunizers***

With respect to subjective norms, Belgian and German immunizers tended to talk to their colleagues about influenza vaccination, while Dutch immunizers largely did not.

“Yes, we are talking about it every year on the ward, when it’s autumn and a flu epidemic is coming”.

Belgian and German HCP reported being uncertain about how many of their colleagues get vaccinated against influenza, while Dutch HCP thought that the majority of their colleagues would also get immunized.

“In my view, most people go get it (influenza vaccination). They are in favour of it, yes”.

Talking to family members about influenza vaccination was common among all immunizers.

“Exactly, I also encourage my family to get vaccinated, because I think that there is also a heightend risk for flu because of me”.

#### ***Subjective norms of non-immunizers***

Of the non-immunizers, Belgian and Dutch HCP reported to not talk to their colleagues about influenza vaccination or only very superficially, whereas German HCP partially talk to their colleagues about it.

“Geez, coincidently it is talked about (influenza vaccination), but it isn’t gone into much. No, little attention is paid to that”.

Nevertheless, the impression of most non-immunizers was that most colleagues do not get vaccinated against influenza either.

“I think that a big part – at least of my colleagues – is against it, yes”.

Talking with family members was only common among Belgian non-immunizers. Some Belgian participants mentioned that family members did not get vaccinated either.

#### ***Perceived behavioural control of immunizers***

With respect to perceived behavioural control, most immunizers reported to feel that it is a free choice whether to get vaccinated against influenza. However, the perception that it is part of an occupational obligation and that there is some pressure to get vaccinated (i.e., from the occupational physician), made the decision not entirely free for some immunized HCP.

“Er yes, but you also have a responsibility in a hospital for your patient population. So there’s a chance there will be pressure to do it from outside, because of that and as long as it doesn’t involve problems for me – that I cannot tolerate it or something – yes okay, then it is part of my job to have mandatory vaccinations”.

#### ***Perceived behavioural control of non-immunizers***

German and Dutch non-immunizers reported the same perception with regard to their freedom to choose whether to get vaccinated as immunizers, whereas all Belgian non-immunizers reported feeling completely free to choose. Freedom of choice was reported to be important for HCP.

“Yes, it has to be my own decision. I just heard that you can be obligated in particular work environments. But I don’t think that that’s okay. It has to be out of free will. Yes”.

### Responsibility

#### ***Responsibility of immunizers***

The majority of immunizers reported that they feel responsible as HCP to protect patients by getting vaccinated against influenza.

“When I walk around here being ill with the flu, it’s also not good for our patients, of course. I mean, the people are already ill and when they then get flu in addition to that, that’s never good”.

Some German HCP reported that vaccination is not the only means of protection, respectively that responsibility for patients can also be taken through other measures.

“Yes, but we otherwise have to wear a mask. […] For me it doesn’t make a difference if I’m vaccinated or if I wear a mask. Patients are protected anyway”.

Direct patient contact and wanting to prevent work absenteeism were reported to additionally increase feelings of responsibility.

#### ***Responsibility of non-immunizers***

In contrast to the immunizers, many non-immunizers did not feel responsible to get vaccinated against influenza to protect patients. Reasons for not feeling responsible to protect patients by getting vaccinated were the belief that regular hand disinfection and staying at home when ill are equally or more effective means of protecting patients against influenza.

“Responsibility for patients, to get vaccinated? Against the flu? No, because when I have it (flu), I stay at home. I take that responsibility”.

Moreover, it was reported that vaccination would not guarantee the protection of patients and can therefore not be the responsibility of HCP and that patients would still be susceptible to other bacteria or viruses that HCP cannot protect their patients from.

“It is my responsibility to protect patients. But being vaccinated or not, I can still get the flu. So you don’t solve anything with a vaccine. So in terms of that, I don’t feel responsible for it”.

Moreover, a lack of reciprocity, or that patients should be equally accountable to protect HCP by getting vaccinated decreased feelings of responsibility. One participant concluded that it is not the responsibility of HCP to get vaccinated for patient safety, because it would otherwise be mandatory in hospitals.

### Experiences with the flu shot

#### ***Experiences of immunizers***

The majority of immunizers reported positive experiences with the flu shot in the past, with - if anything - local pain at the site of injection as a negative aspect.

“When I had something it was local, that you have a local pain for one or two days. But I think you have that with every vaccination”.

Few participants reported the experience of mild side-effects, such as having a cold after getting vaccinated against influenza.

#### ***Experiences of non-immunizers***

Many non-immunizers never got vaccinated against influenza before. The experiences of those that had been vaccinated before differed. Some experiences were positive, while some non-immunizers reported that they had experienced side-effects, ranging from a fever to flu-like symptoms and upper respiratory infections.

“Ahem, I got vaccinated on duty and then I was ill for two days, even though I maybe got ill once in 25 years. So it was actually a negative experience. That’s why I say no for the time being”.

Some non-immunizers had gotten flu irrespective of being vaccinated or not and therefore said that they did not experience a difference with or without influenza vaccination.

### Motivation and barriers

#### ***Motivation and barriers of immunizers***

The intention to get vaccinated again was reported by the majority of immunized HCP. Possible barriers of converting this intention into action were mostly organizational issues, such as time pressure and administrative barriers that might prevent them from getting the flu shot.

“Well, that’s actually mostly in terms of planning. Or at that moment, I know that last time, fortunately there was a second round or something. Because I think I was on duty and then, or there were very specific hours and we had a meeting…Simply in terms of planning”.

Being ill at the time that vaccination appointments are offered and fear of side-effects were also mentioned as barriers.

“At the time that you are ill, you are not allowed to get it (flu shot) I think. But that’s the only one (barrier) I think”.

One misconception that was reported by some immunizers was that pregnancy would be a barrier against getting vaccinated.

“Pregnant women. I don’t know for sure, I would have to look up if they are allowed to get vaccinated or not”.

#### ***Motivation and barriers of non-immunizers***

Among Belgian and Dutch non-immunizers, most did not intend to get vaccinated against influenza in the next season, whereas this was rather divided among German HCP.

“No, I’m not planning on doing it. No”.

Organizational issues were also reported barriers by non-immunizers, together with forgetfulness, illness, and fear of side-effects.

“Well, what I said, because you don’t know how you will react to the vaccination”.

### Patient advice

#### ***Patient advice of immunizers***

Participants were asked if they would recommend influenza vaccination to their patients. German and Dutch immunizers mostly reported that they would recommend influenza vaccination to their patients, especially if they belong to the risk group.

“I don’t recommend it to every patient, patients that have a heightend risk: immunocompromised patients, patients with lung diseases, patients aged 65 years and older. To them I do recommend it strongly”.

Only some Belgian immunizers would recommend the flu shot. The belief that vaccination is a free decision and that one can only inform people about the benefits, but that it is the responsibility and free choice of the patients was present among most immunizers.

“Not really my responsibility. They still decide that themselves. But you can tell your opinion and the advice, the recommendation to do it”.

Time constraints and thinking that it is the task of the responsible practitioner to recommend vaccination were additional reasons to not advice patients about influenza vaccination. All immunized physicians reported to advice influenza vaccination to patients belonging to the risk group, while some nurses said that they would not recommend it.

#### ***Patient advice of non-immunizers***

In many cases, non-immunizers would also recommend influenza vaccination, if their patients belong to the risk group.

“Yes, only the patients that really need it, such as people with a cardiac valve, weak condition, those I would (recommend it), the elderly, yes”.

However, some non-immunizers said that they would not recommend vaccination.

“No I wouldn’t. But that’s just because the topic isn’t that present in my head, that I would go and address it during my work”.

Duties that were perceived as being more important and not being the responsible practitioner were again reasons to not advice vaccination. Non-immunized physicians were in general more willing to advice patients about vaccination than nurses.

## Discussion

The aim of this study was to explore the social cognitive variables and beliefs associated with the decision to get vaccinated of HCP in Belgium, Germany and the Netherlands with interviews to gain a direct and more in-depth understanding of these determinants and to identify beliefs that have not been captured by previous quantitative studies.

Belgian, Dutch and German immunizers all reported self-protection, patient protection, and protection of family members as most important reasons to get vaccinated against influenza. It has been suggested before, that the realistic assessment of potential benefits of influenza vaccination for especially the self, but also for others are crucial motivating factors for HCP to get vaccinated [25,26]. In contrast to that, Belgian, Dutch and German non-immunizers’ reasons to not get vaccinated against influenza can be clustered into six categories: 1) fear of side effects or illness caused by the vaccine, 2) a low risk-perception, 3) the disbelief in the effectiveness of influenza vaccination, 4) organizational barriers, 5) misconceptions/lack of knowledge, and 6) undefined negative emotions or fear resulting from the decision whether to get vaccinated. These reasons to not get vaccinated are in line with findings from previous empirical and review studies [17-20,26-28].

The attitude towards influenza vaccination was generally positive, however outcome expectations seemed to influence the decision whether to get vaccinated. With regard to subjective norms, HCP might be less influenced by what they talk about with colleagues (injunctive norm), than by what they think that colleagues would do (descriptive norm). HCP that did get vaccinated themselves had the impression that most of their colleagues also get vaccinated, while the opposite was true for non-immunizers. In contrast, Looijmans-van den Akker and colleagues [27] had found a significant association of injunctive subjective norms with vaccination uptake. Moreover, immunized HCP had more and better experiences with the flu shot, felt more responsible to protect patients by getting vaccinated and intended to get vaccinated against influenza again. Previous influenza vaccination uptake had been shown to be a main predictor of future vaccination uptake [17,18]. For some non-immunizers, feelings of responsibility to protect patients seemed to be associated with feelings of reciprocity, or the expectation that patients should be equally accountable to protect HCP by getting vaccinated. Reciprocity has been suggested to be an important factor influencing altruistic behaviour [29,30].

With respect to perceived behavioural control, most HCP reported to feel that it is a free choice whether to get vaccinated against influenza. Freedom of choice has been shown to be highly valued even by HCP that get vaccinated against influenza [25].

Among the reasons that influenced the decision to not get vaccinated, we identified three additional beliefs that have not been extensively described in the literature before. Firstly, a reason to not get vaccinated was the belief that other means of prevention, such as regular hand disinfection or staying at home when ill are as effective or even more effective in preventing influenza transmission to patients than influenza vaccination [31]. Secondly, some immunizers showed an omission bias, which is the preference to not get vaccinated over getting vaccinated if one thinks that vaccination could cause illness [32]. The omission bias was previously associated with parent’s decision to not vaccinate their children [33]. Finally, other health beliefs surfaced that comprise the belief that it is better for one’s health to undergo illness and to build own antibodies during illness than to prevent illness by getting vaccinated, which is related to a previously described belief that vaccination would weaken the immune system [28].

Furthermore, HCP recommend influenza vaccination to patients, when they belong to the risk group. However, vaccination was seen as a free choice, not only for HCP, but also for patients. The question was hypothetical in nature and it seemed that immunizers, as well as non-immunizers were very rarely asked about influenza vaccination by patients and did not talk about vaccination with their patients due to other topics and duties that were perceived as more important. Moreover, it was reported that only authorized practitioners would be allowed to give advice about medication and vaccination, which means that it is not a part of the duties of most of the participants in this study. Accordingly, more immunized and non-immunized physicians were willing to advice influenza vaccination to patients than immunized and non-immunized nursing staff.

This study extends current knowledge about social cognitive variables and beliefs that affect the motivation of HCP to get vaccinated against influenza. The study was conducted in three European countries, one of them not having been systematically surveyed before (Belgium). The present study used a qualitative research method, which has the advantage of gaining a direct and in-depth understanding of the beliefs underlying the decision to get vaccinated against influenza of HCP [21]. However, due to cross-sectional analysis no causal relationships could be established, nor the relative importance of social cognitive variables and beliefs in explaining why HCP get or do not get vaccinated against influenza. Future research should use the insights of this study and quantify the results. Moreover, future studies should explore the predictive value of the social cognitive variables and beliefs found in this study in explaining the intention to get vaccinated. Secondly, coding of the interviews was performed by only one coder, which made inter-rater reliability not possible. This could have biased the results. Qualitative research is inherently interpretive and more coders could potentially decrease bias. However, coding was done in a systematic way by developing a coding scheme and all authors discussed the analysis process and interpretation of the data extensively so as to reduce bias to a minimum. Thirdly, few participants belonged to a high risk group (N = 5), due to age, pregnancy or a medical condition. We did not exclude them, however it should be noted that their reasons for getting vaccinated against influenza could be related to their condition, rather than their occupation. Finally, the participating hospitals were chosen based on convenience, rather than representativeness of hospitals in the three different countries. Therefore, generalizations to the collective population of Belgian, Dutch and German HCP should be treated with caution.

## Conclusions

The reasons that HCP have for getting vaccinated against influenza (or not) seem to be similar in Belgium, Germany and the Netherlands. This was also true for the social cognitive variables that are believed to drive the intention to get vaccinated, even though some differences surfaced. A quantitative investigation of those social cognitive variables is needed in order to determine the importance of the social cognitive variables in explaining the intention to get vaccinated and the importance of the similarities and differences between countries that have been found in this study. This would in turn shed more light onto the question whether intervention programs developed to increase vaccination uptake, have to be country-specific or if one intervention program can be used in different countries.

## Abbreviations

HCP: Healthcare personnel.

## Competing interests

All authors declare that they have no competing interests.

## Authors’ contributions

BAL, RACR, and GK contributed to the conception, design of the study, as well as the interpretation of the data. BAL acquired the data and undertook analysis. All authors contributed to drafting the paper and read and approved the final manuscript.

## Pre-publication history

The pre-publication history for this paper can be accessed here:

http://www.biomedcentral.com/1471-2458/14/407/prepub

## Supplementary Material

Additional file 1 The RATS checklist. Click here for file
