# Supplementary material for: “I don’t see an added value for myself”: a qualitative study exploring the social cognitive variables associated with influenza vaccination of Belgian, Dutch and German healthcare personnel
Source: BMC Public Health. 2014 Apr 28;14:407. doi: 10.1186/1471-2458-14-407 (PMC4021212; doi:10.1186/1471-2458-14-407)
Supplement: Additional file 1 — The RATS checklist. [file 1471-2458-14-407-S1.docx]

| **ASK THIS OF THE MANUSCRIPT** | **THIS SHOULD BE INCLUDED IN THE MANUSCRIPT** | **Reference in manuscript** |
| --- | --- | --- |
| **R Relevance of study question** |  |  |
| Is the research question interesting? | Research question explicitly stated | The research question is stated at the end of the background-section (p.4-5). |
| Is the research question relevant to clinical practice, public health, or policy? | Research question justified and linked to the existing knowledge base (empirical research, theory, policy) | The background justifies the research question by outlining the existing knowledge base (p.3-4). |
| **A Appropriateness of qualitative method** |  |  |
| Is qualitative methodology the best approach for the study aims?   - *Interviews:* experience, perceptions, behaviour, practice, process - *Focus groups:* group dynamics, convenience, non-sensitive topics - *Ethnography:* culture, organizational behaviour, interaction - *Textual analysis:* documents, art, representations, conversations | Study design described and justified i.e., why was a particular method (e.g., interviews) chosen? | Approach (one-to-one interviews) described and justified (p.4). |
| **T Transparency of procedures** |  |  |
| *Sampling* |  |  |
| Are the participants selected the most appropriate to provide access to the type of knowledge sought by the study?  Is the sampling strategy appropriate? | Criteria for selecting the study sample justified and explained   - *theoretical:* based on preconceived or emergent theory - *purposive:* diversity of opinion - *volunteer:* feasibility, hard-to-reach groups | The included sample is a hard-to-reach group. We tried to obtain a comparable proportion of physicians and nursing staff in the sample. Participation was voluntary. This is described and justified (p.5). |
| *Recruitment* |  |  |
| Was recruitment conducted using appropriate methods? | Details of how recruitment was conducted and by whom | Possible participants were approached during their lunch breaks or in the waiting room of the occupational physician. Recruitment was performed by the first author (BAL) and continued until saturation occurred (p.5). |
| Is the sampling strategy appropriate? |  |  |
| Could there be selection bias? | Details of who chose not to participate and why | Participation was open to all healthcare personnel (p.5). |
| *Data collection* |  |  |
| Was collection of data systematic and comprehensive? | Method(s) outlined and examples given (e.g., interview questions) | Methods are outlined and examples of interview questions are given (p.6). |
| Are characteristics of the study group and setting clear? | Study group and setting clearly described | Study group and setting are clearly described (p.5). |
| Why and when was data collection stopped, and is this reasonable? | End of data collection justified and described | Data collection was continued until saturation occurred, as described in Patton MQ: *Qualitative evaluation and research methods.* 2nd edition. Newbury Park: Sage Publications; 1990. (p.5). |
| *Role of researchers* |  |  |
| Is the researcher(s) appropriate? How might they bias (good and bad) the conduct of the study and results? | Do the researchers occupy dual roles (clinician and researcher)? Are the ethics of this discussed? Do the researcher(s) critically examine their own influence on the formulation of the research question, data collection, and interpretation? | Researcher role described in the methods (no dual relationship with interviewees) (p.5-6) |
| *Ethics* |  |  |
| Was informed consent sought and granted? | Informed consent process explicitly and clearly detailed | See the description in the methods (p.5). |
| Were participants’ anonymity and confidentiality ensured? | Anonymity and confidentiality discussed | All quotes were anonymised. Data is stored confidentially. |
| Was approval from an appropriate ethics committee received? | Ethics approval cited | The Research Ethics Board of the Faculty of Psychology and Neuroscience at Maastricht University reviewed and approved the study. |
| **S Soundness of interpretive approach** |  |  |
| *Analysis* |  |  |
| Is the type of analysis appropriate for the type of study?   - *thematic:* exploratory, descriptive, hypothesis generating - *framework:* e.g., policy - *constant comparison/grounded theory:* theory generating, analytical   Are the interpretations clearly presented and adequately supported by the evidence? | Analytic approach described in depth and justified  *Indicators of quality:* Description of how themes were derived from the data (inductive or deductive)  Evidence of alternative explanations being sought  Analysis and presentation of negative or deviant cases | Analytical approach described on p.6.  The content analysis was based on a combination of a deductive and a general inductive approach.  Interpretations are clearly presented and supported by evidence (see results). |
| Are quotes used and are these appropriate and effective? | Description of the basis on which quotes were chosen  Semi-quantification when appropriate  Illumination of context and/or meaning, richly detailed | Quotes were selected on the basis of their representativeness for the findings and context and meaning are described (results section). |
| Was trustworthiness/reliability of the data and interpretations checked? | Method of reliability check described and justified e.g., was an audit trail, triangulation, or member checking employed? Did an independent analyst review data and contest themes? How were disagreements resolved? | Content analysis was conducted by a single coder. No formal testing of reliability could be performed. However, coding was done in a systematic way by developing a coding scheme and all authors discussed the analysis process and interpretation of the data extensively. |
| *Discussion and presentation* |  |  |
| Are findings sufficiently grounded in a theoretical or conceptual framework?  Is adequate account taken of previous knowledge and how the findings add? | Findings presented with reference to existing theoretical and empirical literature, and how they contribute | Findings presented with reference to existing theoretical and empirical literature, and how they contribute. |
| Are the limitations thoughtfully considered? | Strengths and limitations explicitly described and discussed | Strengths and weaknesses are covered in the discussion (p.20-21). |
| Is the manuscript well written and accessible? | Evidence of following guidelines (format, word count)  Detail of methods or additional quotes contained in appendix  Written for a health sciences audience | The manuscript follows the guidelines of BMC Public Health and was written for a health science audience. Details of participant’s responses depicted in Table 2. |
| Are red flags present? These are common features of ill-conceived or poorly executed qualitative studies, are a cause for concern, and must be viewed critically. They might be fatal flaws, or they may result from lack of detail or clarity. | *Grounded theory:* not a simple content analysis but a complex, sociological, theory generating approach  *Jargon:* descriptions that are trite, pat or jargon filled should be viewed sceptically  *Over interpretation:* interpretation must be grounded in "accounts" and semi-quantified if possible or appropriate  *Seems anecdotal, self evident:* may be a superficial analysis, not rooted in conceptual framework or linked to previous knowledge, and lacking depth  *Consent process thinly discussed:* may not have met ethics requirements  *Doctor-researcher:* consider the ethical implications for patients and the bias in data collection and interpretation | No red flags were identified by the authors of this manuscript or reviewers. |
